# Health Care Contact Days and Outcomes in Clinical Trials vs Routine Care Among Patients With Non–Small Cell Lung Cancer

**DOI:** 10.1001/jamanetworkopen.2025.5033

**Published:** 2025-04-15

**Authors:** Arjun Gupta, Paul Nguyen, Brooke E. Wilson, Christopher M. Booth, Timothy P. Hanna

**Affiliations:** 1 Division of Hematology, Oncology, and Transplantation, University of Minnesota, Minneapolis; 2 ICES Queen’s, Queen’s University, Kingston, Ontario, Canada; 3 Division of Cancer Care and Epidemiology, Sinclair Cancer Research Institute, Kingston, Ontario, Canada; 4 Department of Oncology, Queen’s University, Kingston, Ontario, Canada; 5 Department of Medicine, Queen’s University, Kingston, Ontario, Canada

## Abstract

**Importance:**

Although patients enrolled in trials have superior survival outcomes compared with those in routine practice, it is unknown whether such differences extend to contact days, a measure of time toxicity.

**Objective:**

To evaluate differences in contact days for patients with advanced stage non–small cell lung cancer (NSCLC) receiving care in trials or routine practice.

**Design, Setting, and Participants:**

This population-based, retrospective, matched cohort study assessed adults from Ontario, Canada, who were diagnosed with advanced-stage NSCLC between January 1, 2010, and December 31, 2017, and who died between January 1, 2010, and December 31, 2019. The maximum follow-up time from diagnosis was 2 years. Data analysis was performed from May 5, 2024, to October 22, 2024.

**Exposure:**

Patients receiving specific, systemic, palliative-intent, cancer-directed drug(s) as part of a trial were matched 1:1 with patients who received the same drug(s) after approval in routine practice in the same line of treatment.

**Main Outcomes and Measures:**

Contact days (days with in-person health care contact) were identified through administrative claims data. Models were fitted with cubic splines to describe trajectories of weekly percentage of contact days.

**Results:**

Of the 250 patients (mean [SD] age, 63.6 [9.2] years; 140 [56.0%] male), 125 were trial participants and 125 were receiving care in routine practice. Trial participants were younger (median [IQR] age, 63 [56-69] years vs 64 [58-70] years in routine care patients; standardized difference, 0.21) and had fewer comorbidities (eg, hypertension [45 (36.0%) vs 59 (47.2%); standardized difference, 0.23]). Median (IQR) contact days from diagnosis to death were higher for trial participants compared with those in routine practice (79 [62-104] vs 68 [46-98] days; standardized difference, 0.26). However, trial participants had a longer median (IQR) overall survival (eg, 12.8 [8.7-18.0] vs 10.5 [5.2-14.7] months; standardized difference, 0.46) and a slightly lower median percentage of contact days after adjusting for survival (20.3% [95% CI, 18.1%-21.7%] vs 21.2% [95% CI, 19.3%-25.7%]). During treatment, trial participants experienced a lower median percentage of contact days (18.4% [95% CI, 16.3%-20.8%] vs 25.5% [95% CI, 20.7%-30.3%]); inpatient care accounted for 18.5% (95% CI, 11.1%-29.6%) of on-treatment contact days for trial participants vs 40.0% (95% CI, 30.0%-47.6%) in routine practice. Normalized contact-day trajectories were U-shaped for all groups, with lower peaks and troughs among trial participants.

**Conclusions and Relevance:**

In this population-based cohort study, patients receiving systemic therapy as part of trials experienced a lower percentage of contact days, accounted for by greater hospitalization rates in routine practice. Addressing the predominantly outpatient, protocol-mandated visits may represent opportunities to decrease trial-related time toxicity.

## Key Points

**Question:**

Were there differences in health care contact days between patients with advanced-stage non–small cell lung cancer treated in clinical trials vs routine practice?

**Findings:**

In this cohort study of 250 patients, those who received the same systemic cancer-directed drug(s) in the same line of treatment within the same health system in clinical trials experienced a lower proportion of contact days during active treatment.

**Meaning:**

The greater proportion of contact days (higher time toxicity) during active treatment for patients in routine practice has ramifications for setting expectations for these patients, while easing theoretical concerns regarding the additional time toxicity associated with clinical trial participation.

## Teaser

This cohort study examines patterns of contact days among patients with advanced-stage non–small cell lung cancer receiving care in clinical trials compared with routine practice.

## Introduction

Time toxicity, the amount of time spent by patients receiving cancer care, is an increasingly studied concept.^1^ Patients with advanced-stage solid cancers spend a large proportion of their days alive with health care contact (ie, contact days). Multiple retrospective studies across health systems and countries, including patients treated as part of early-phase trials, later-phase trials, and in routine practice, have reported an approximately 20% to 33% rate of contact days.^2,3,4,5,6,7,8,9^ A key emerging question is how patterns of contact days vary between patients receiving the same treatment(s) in trials vs routine practice.^10^ There is a clear efficacy-effectiveness (EE) gap when it comes to survival, with patients in routine practice often having inferior survival outcomes compared with those in trials.^11,12^ However, whether this EE gap extends to time toxicity is unknown. Although patients in trials face protocol-mandated visits (and resultant planned contact days) over and above what would be expected in routine clinical care,^13^ patients in routine practice often have more comorbidities and less fitness and experience higher rates of physical toxic effects (and resultant unplanned contact days).^1^ Evaluating this potential time toxicity EE gap is important because it may allow for care improvement opportunities in both settings and clarify whether trial data could set expectations about time toxicity in routine practice.

Although clinical need (patient, cancer, and treatment characteristics) is an important determinant of contact days, health system factors (eg, care access) can also influence contact days. An ideal evaluation of the time toxicity EE gap would have both comparison groups (trial and routine practice) receiving care in the same health system. This evaluation is particularly feasible and relevant in disease sites with recent investigation, approval, and uptake of cancer-directed treatments, such as advanced-stage non–small cell lung cancer (NSCLC).^14^ We thus sought to characterize the time toxicity EE gap by evaluating patterns of contact days among patients with advanced-stage NSCLC receiving the same cancer-directed drug treatment(s) within and outside trials in a large population-based cohort.

## Methods

### Setting and Case Selection

We created a population-based, retrospective cohort of adults 20 years or older with stage III and IV (American Joint Committee on Cancer, 7th and 8th editions) NSCLC receiving palliative systemic cancer-directed drug(s) as part of a trial, using administrative data collected through the Ontario Cancer Registry (eTable 1 in Supplement 1). The study was approved by the Queen’s University Health Sciences and Affiliated Teaching Hospitals Research Ethics Board and followed Strengthening the Reporting of Observational Studies in Epidemiology (STROBE) and Reporting of Studies Conducted Using Observational Routinely Collected Data (RECORD) reporting guidelines.^15,16^ In accordance with these guidelines, the following sections and eTable 2 in Supplement 1 describe data sources and linkage, and a flow diagram describing the study cohort is provided in eFigure 1 in Supplement 1. A waiver of informed consent was granted based on the Personal Health Information Protection Act.

Ontario has a population of 16.1 million, approximately 39% of the Canadian population, and provides universal health care coverage to all eligible residents.^17^ We identified patients diagnosed with NSCLC from January 1, 2010, to December 31, 2017, who died from January 1, 2010, to December 31, 2019. This period captured a time of intense investigation and drug approval in NSCLC, allowed time for adoption in clinical practice, and avoided care disruption due to the COVID-19 pandemic. We identified trial participation with the Activity Level Reporting database, a patient-level system that contains systemic delivery records of cancer-directed and supportive medications (including National Clinical Trial [NCT] identification).

We directly matched patients receiving systemic cancer-directed drug(s) as part of a clinical trial 1:1 with those who received the same drug(s) in routine practice (after approval) in the same line of treatment. We did not match on any other features because we considered these patient, system, and disease factors as mediators of the hypothesized EE gap. For example, there could be differences in prior or subsequent lines of treatment. Due to privacy regulations, we are unable to report the specific drugs.^18^ When the NCT identifier was available in the treatment record, we extracted publicly available trial characteristics from the ClinicalTrials.gov website.

### Data Sources and Linkage

Data were obtained from administrative datasets (eTable 2 in Supplement 1) housed at ICES, formerly known as the Institute for Clinical Evaluative Sciences, an independent, nonprofit research institute funded by the Ontario Ministry of Health and the Ministry of Long-Term Care. Cancer-specific data were abstracted from the Ontario Cancer Registry, a population-based tumor registry administered by Ontario Health. The registry passively collects cancer data on Ontario residents through pathology reporting, hospital records, treatment centers, and death records. Systemic therapy in trials was abstracted from the Activity Level Reporting database. Supplementary information on systemic therapy for routine practice was identified with the New Drug Funding Program and Ontario Drug Benefit program. These datasets were linked using unique encoded identifiers and analyzed at ICES.

### Covariates

We extracted sociodemographic and clinical characteristics (eTable 2 in Supplement 1), including age, sex, income, place of residence, rurality of residence, comorbidities, year of cancer diagnosis, cancer anatomical location, and histologic and morphologic findings. Data on race and ethnicity were not available in the source data for our cohort. Comorbidities were measured using the Elixhauser comorbidity index derived from hospital records with a 5-year lookback from their NSCLC diagnosis. Chronic conditions were extracted from the ICES-derived databases. The shortest driving distance and duration from place of residence to the nearest regional cancer center (obtained from Ontario Health) were estimated using the Open Source Routing Machine API with OpenStreetMap.^19^

### Exposures and Outcomes

The primary exposure was receipt of treatment on trial vs in routine practice. We further categorized patients into subgroups based on (1) the line of treatment in which the relevant drug(s) were received and (2) the type of drug(s) received (cytotoxic chemotherapy, immunotherapy only, or targeted therapy only). The primary outcome was the median percentage of health care contact days (days with health care contact outside the home for any length of time or reason).^1,20^ Overall survival was the number of days from stage III to IV NSCLC diagnosis to death.

Contact days were classified into inpatient (inpatient acute care or rehabilitation hospitalizations, emergency department visits, or long-term or complex continuing care) and outpatient (eg, family physician and cancer clinic visits, blood tests, imaging, outpatient operations, dialysis, injections or infusions, and radiotherapy assessment and treatments). The following blood tests were included: red blood cell, white blood cell, platelet, bilirubin, potassium, thyrotropin, creatinine, or glycated hemoglobin. If both inpatient and outpatient care occurred on a day, it was only considered an inpatient contact.

Vital status was censored 2 years from diagnosis, with the latest date of follow-up being December 31, 2019. We considered 2 secondary study periods: (1) on-treatment period (time from first administration of the relevant drug[s] to the expected end of cycle [date of last administration + cycle length]) and (2) pretreatment period (for the first-line subgroup, the 30-day period before the initiation of the relevant drug[s]). We calculated time from diagnosis to initiation of systemic therapy.

### Statistical Analysis

We generated summary statistics for baseline characteristics. Median percentage of contact days was estimated as the median number of contact days divided by median overall survival or median time receiving treatment. To measure the uncertainty of the median percentage of contact days, we used bootstrapping to compute the nonparametric percentile-based CIs with 10 000 samples.^21^ We conducted subgroup analyses for patients who received relevant drug(s) in the first line. We were unable to conduct some subgroup analyses (eg, immunotherapy) due to low numbers.

We plotted the percentage of weekly contact days (percentage of contact days in each week) from diagnosis to death. To facilitate visualization of trajectories for patients with differential survival, we rescaled (minimum-maximum normalization) the survival time and fitted a cubic smoothing spline to the normalized observations. We additionally created plots for inpatient and outpatient contact days. To examine patterns of contact days during various phases of care, and specifically during the time on treatment, among patients receiving the relevant drug(s) in the first line, we divided the normalized time into phases (pretreatment, on treatment, and posttreatment) by the mean time spent in each phase. We calculated the area under the modeled curve and expressed it as a percentage of a 1 × 1 area.

Differences in contact days and patient characteristics were assessed with standardized differences using a conservative threshold of greater than 0.2 for detecting effect sizes.^22^ We conducted analyses from May 5, 2024, to October 22, 2024, using SAS software, version 9.4 (SAS Institute Inc).

## Results

### Baseline Characteristics

The study included 250 patients (mean [SD] age, 63.6 [9.2] years; 140 [56.0%] male and 110 [44.0%] female), with 125 receiving systemic cancer-directed drug(s) as part of a trial and 125 receiving the same drugs in the same line in routine practice (eFigure 1 in Supplement 1). Compared with patients in routine practice, trial participants were younger (median [IQR] age, 63 [56-69] years vs 64 [58-70] years in routine care patients; standardized difference, 0.21), had fewer comorbidities (eg, hypertension [45 (36.0%) vs 59 (47.2%); standardized difference, 0.23]), and had shorter estimated mean (SD) driving distances (38.7 [42.0] vs 53.6 [75.8] km; standardized difference, 0.24) (Table 1).

**Table 1.** Sociodemographic and Clinical Characteristics by Patients Receiving Treatment as Part of a Clinical Trial or in Routine Practice for Patients With Non–Small Cell Lung Cancer, January 1, 2010, to December 31, 2017, Ontario, Canada

Characteristic	Patients, No. (%)^a^		Standardized difference
	Clinical trial participants (n = 125)	Patients in routine practice (n = 125)	
Age, median (IQR), y	63 (56-69)	64 (58-70)	0.21^b^
Age group, y			
20-59	50 (40.0)	35 (28.0)	0.26^b^
60-69	47 (37.6)	55 (44.0)	0.13
≥70	28 (22.4)	35 (28.0)	0.13
Sex			
Female	58 (46.4)	52 (41.6)	0.10
Male	67 (53.6)	73 (58.4)	0.10
Income quintile			
1 (Lowest)	27 (21.6)	16 (12.8)	0.23^b^
2	24 (19.2)	28 (22.4)	0.08
3	27 (21.6)	28 (22.4)	0.02
4	22 (17.6)	24 (19.2)	0.04
5 (Highest)	24 (19.2)	29 (23.2)	0.10
Rurality			
Urban (RIO <10)	85 (68.0)	74 (59.2)	0.18
Suburban (RIO 10 to <40)	26 (20.8)	27 (21.6)	0.02
Rural (RIO ≥40)	13 (10.4)	22 (17.6)	0.21^b^
Estimated shortest driving distance to cancer center, mean (SD), km^c^	38.7 (42.0)	53.6 (75.8)	0.24^b^
Estimated shortest driving duration to cancer center, mean (SD), min^c^	35.0 (27.6)	44.1 (49.4)	0.23^b^
Histologic and morphologic findings			
Neoplasm, not otherwise specified, or other	30 (24.0)	23 (18.4)	0.12
Squamous cell neoplasm	20 (16.0)	23 (18.4)	0.06
Adenomas or adenocarcinoma	75 (60.0)	79 (63.2)	0.07
Chronic conditions			
Asthma	15 (12.0)	10 (8.0)	0.13
Chronic obstructive pulmonary disease	34 (27.2)	43 (34.4)	0.16
Hypertension	45 (36.0)	59 (47.2)	0.23^b^

Abbreviation: RIO, Rurality Index for Ontario.

^a^ Unless otherwise indicated. Column percentages may not sum to 100% due to missing data.

^b^ Indicates notable effect sizes. A conservative threshold of greater than 0.2 was used for standardized differences to detect notable effect size between patients treated as part of clinical trials and in routine practice.

^c^ Driving distance and duration were measured with the shortest distance generated from the Open Source Routing Machine API with OpenStreetMap data between the postal code of residence and the geographic location of the regional cancer center.

### Treatment Characteristics

The median (IQR) number of therapy lines received in both groups was 1 (1-2); 72 patients (57.6%) received the relevant drug(s) in the first line. Among the 125 patients in each group, the most common treatment type was cytotoxic chemotherapy (84 [67.2%]). The median (IQR) time from diagnosis to initiation of systemic therapy was 2.1 months in both groups (2.1 [1.5-3.5] months in trial patients vs 2.1 [1.4-3.9] months in routine care patients; standardized difference, 0.02). The NCT identification number was available for 18 trials that enrolled 53 of the 125 trial participants. Of these 18 trials, 8 were phase 3, 10 evaluated cytotoxic chemotherapy, and the median study size was greater than 300.

### Survival and Time Toxicity Outcomes

#### All Patients

**Figure 1.**
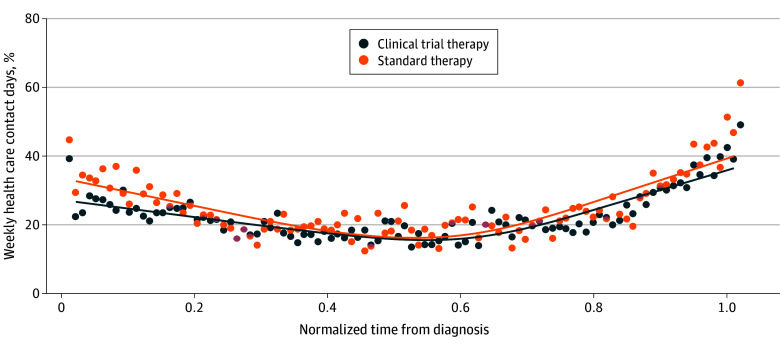
Weekly Contact Days From Diagnosis to Death for Trial Participants and Patients in Routine Practice To ensure a fair comparison of contact day trajectories and to facilitate visualization of contact day trajectories across patients with differential survival durations, irrespective of individual survival lengths, we normalized the time between non–small cell lung cancer diagnosis and death for each decedent. Cubic smoothing splines (line) were fitted to estimate the trajectories of the time series observations. Mean values of time series observations (dots) were also plotted.

Trial participants had a longer median (IQR) overall survival compared with patients in routine practice (12.8 [8.7-18.0] vs 10.5 [5.2-14.7] months; standardized difference, 0.46), of which 79 and 68 days, respectively, were contact days, corresponding to a slightly lower median percentage of contact days for trial participants (20.3% [95% CI, 18.1%-21.7%] vs 21.2% [95% CI, 19.3%-25.7%]). During treatment, trial participants had a much lower median percentage of contact days (18.4% [95% CI, 16.3%-20.8%] vs 25.5% [95% CI, 20.7%-30.3%]). Of these on-treatment contact days, only a median of 18.5% (95% CI, 11.1%-29.6%) were inpatient for trial participants, whereas 40.0% (95% CI, 30.0%-47.6%) were inpatient for patients in routine practice. Details on patterns and sources of contact are presented in Table 2. Figure 1 presents the percentage of weekly contact days for both groups. Both followed a U-shaped normalized trajectory. Visually, the rate of weekly contact days was slightly lower for trial participants, and the area under the fitted curve was 21% for the trial group and 23% for the routine practice group. eFigure 2 in Supplement 1 presents trajectories of contact days for trial participants and patients in routine practice separately and by outpatient and inpatient contact days.

**Table 2.** Patterns and Sources of Contact Days for Patients With Non–Small Cell Lung Cancer Receiving Treatment as Part of a Clinical Trial or in Routine Practice, January 1, 2010, to December 31, 2017, Ontario, Canada

Variable	During disease course		During time receiving treatment		30-Day pretreatment	
	Trial	Routine practice	Trial	Routine practice	Trial	Routine practice
**All comers (n = 125 in each group)**						
Time in period, median (IQR), d (mo)	390 (264-549) (12.8 [8.7-18.0])	321 (159-447) (10.5 [5.2-14.7])	147 (87-203) (4.8 [2.9-6.7])	98 (56-210) (3.2 [1.8-6.9])	NA	NA
Total contact days, median (IQR)	79 (62-104)	68 (46-98)	27 (20-38)	25 (13-43)	NA	NA
Days in period that were contact days, % (95% CI)^a^	20.3 (18.1-21.7)	21.2 (19.3-25.7)	18.4 (16.3-20.8)	25.5 (20.7-30.3)	NA	NA
Inpatient contact days, median (IQR)	20 (10-34)	21 (11-39)	5 (2-13)	10 (4-17)	NA	NA
Contact days in period that were inpatient contact days, % (95% CI)^a^	25.3 (21.4-32.5)	30.1 (24.7-35.4)	18.5 (11.1-29.6)	40.0 (30.0-47.6)	NA	NA
**First line (n = 72 in each group)**						
Time in period, median (IQR), d (mo)	366 (225-531.5) (12.0 [7.4-17.5])	248 (121-419.5) (8.2 [4.0-13.8])	115 (53-146) (3.8 [1.7-4.8])	67 (40-94.5) (2.2 [1.3-3.1])	30 (30-30) (1.0 [1.0-1.0])	30 (30-30) (1.0 [1.0-1.0])
Total contact days, median (IQR)	77.5 (54-95)	56 (42-96)	22 (15.5-30)	18 (9.5-27)	6 (4.5-8)	6 (4-10)
Days in that period that were contact days, % (95% CI)^a^	21.2 (18.2-25.0)	22.6 (18.6-32.8)	19.1 (15.9-25.6)	26.9 (19.0-32.1)	20.0 (16.7-23.3)	20.0 (13.3-26.7)
Inpatient contact days, median (IQR)	22 (10-33)	19 (11-42)	4 (1-11)	9 (3.5-15)	2.5 (1-6)	6 (2-10)
Contact days in period that were inpatient contact days, % (95% CI)^a^	28.4 (18.8-33.8)	33.9 (22.7-42.6)	18.2 (12.8-36.4)	50.0 (37.5-69.6)	41.7 (16.7-90.0)	100.0 (50.0-100.0)

Abbreviation: NA, not applicable (given factors influencing contact days could be different between lines of therapy, 30-day pretreatment contact days is not presented for all comers).

^a^ Nonparametric percentile-based CIs were obtained from bootstrapping with 10 000 samples.

#### Patients in the First Line

**Figure 2.**
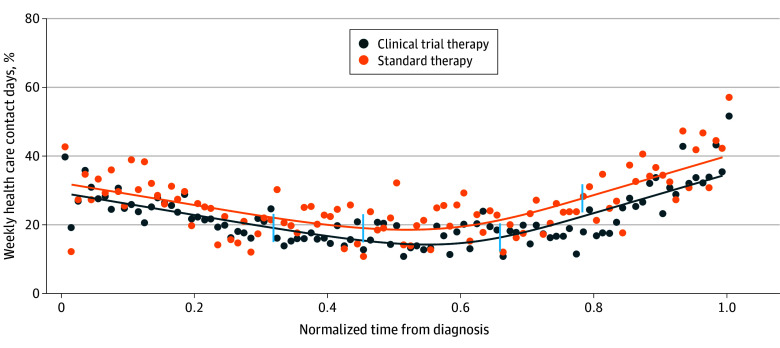
Weekly Contact Days From Diagnosis to Death for Trial Participants and Patients in Routine Practice Getting Relevant Drug(s) in First Line Only and Receiving Only 1 Line of Therapy To ensure a fair comparison of contact day trajectories and to facilitate visualization of contact day trajectories across patients with differential survival durations, irrespective of individual survival lengths, we normalized the time between non–small cell lung cancer diagnosis and death for each decedent. Cubic smoothing splines (line) were fitted to estimate the trajectories of the time series observations. Mean values of time series observations (dots) were also plotted. Vertical ticks on the splines represent the mean of the normalized times from diagnosis to first and last administrations of systemic therapy, respectively. The period between the ticks represents the mean normalized time on treatment.

**Figure 3.**
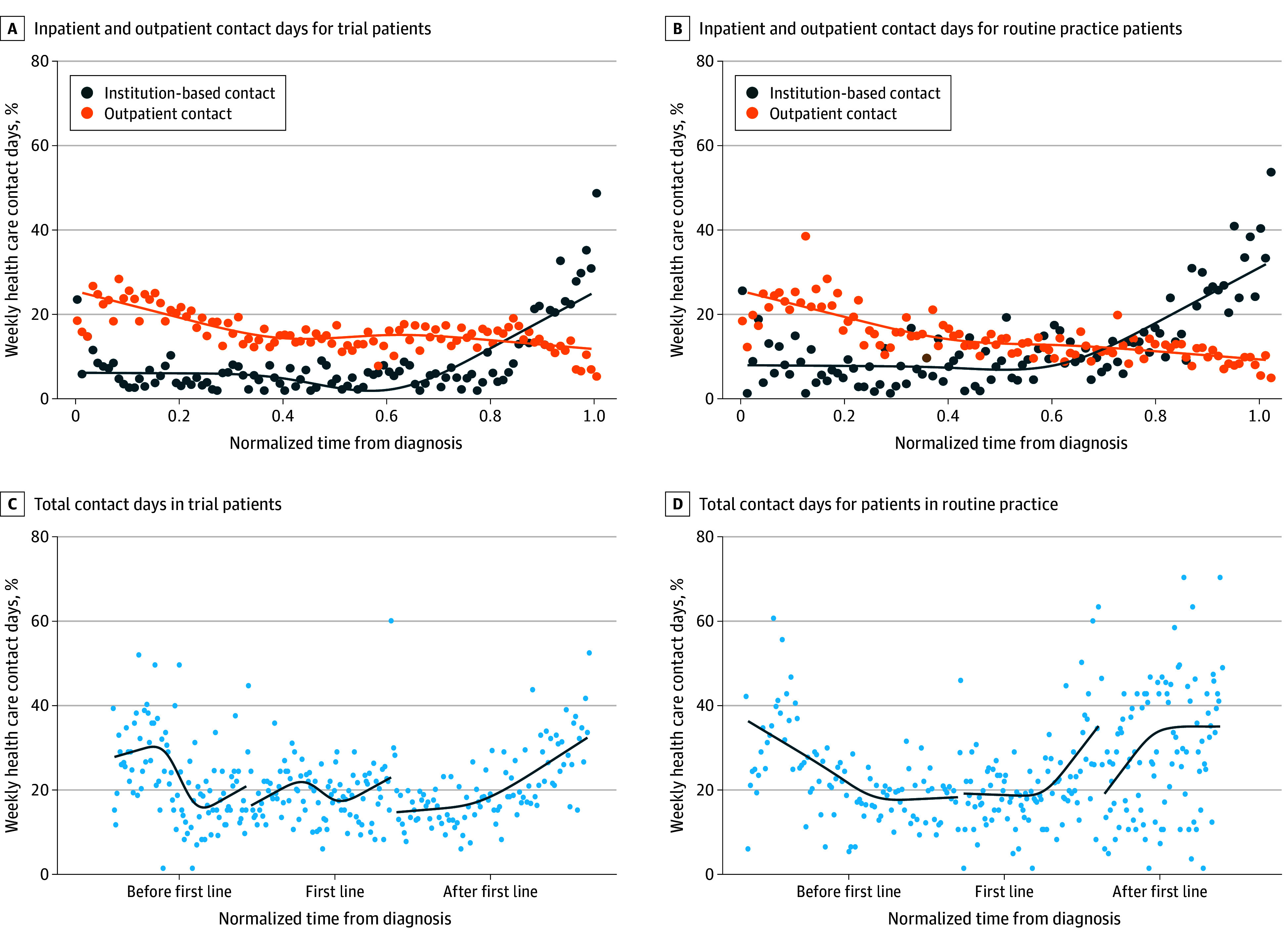
Weekly Contact Days From Diagnosis to Death for Trial Participants and Patients in Routine Practice Getting Relevant Drug(s) in First Line Only For panels C and D, the time during each phase for each individual was normalized.

Trial participants had a significantly longer median (IQR) overall survival (12.0 [7.4-17.5] vs 8.2 [4.0-13.8] months; standardized difference, 0.53), of which 78 and 56 days, respectively, were contact days, corresponding to a lower percentage (95% CI) of contact days throughout the disease course for trial participants (21.2% [18.2%-25.0%] vs 22.6% [18.6%-32.8%]). The median (IQR) time on treatment was longer for trial participants (3.8 [1.7-4.8] vs 2.2 [1.3-3.1] months; standardized difference, 0.54), and they experienced a lower median percentage (95% CI) of contact days during this time (19.1% [15.9%-25.6%] vs 26.9% [19.0%-32.1%]) (Table 2). Figure 2 presents the trajectory of weekly contact days; the area under the fitted curve was lower (20% vs 25%) for the trial group. Trial participants had much lower rates of inpatient days, especially during the middle half of the disease course (Figure 3A and B). During treatment, trial participants had a minisinusoidal pattern of contact days, but the amplitude of variation was low and hovered around 20%, whereas patients in routine practice had a sharp increase in contact days during the second half (Figure 3C and D). Both groups had a 20% contact day rate in the 30-day pretreatment period.

## Discussion

In this contemporary study of trial participants with advanced-stage NSCLC matched to patients in routine practice receiving the same drug(s) in the same line of treatment within the same health care system, we found evidence of a time toxicity EE gap. In line with prior work,^11,12^ we observed a large survival EE gap, but trial participants had a slightly lower rate of contact days throughout the disease course. The time toxicity EE gap was most prominent during the time receiving treatment, when patients in routine practice experienced higher rates of inpatient days. Encouragingly, trial participants did not experience delays in treatment initiation and did not experience higher rates of time toxicity, potentially allaying some concerns regarding trial participation. However, trial participants did experience an approximate 20% contact day rate, comprising mostly outpatient contact days. These results provide the first evidence of a time toxicity EE gap, with ramifications for setting expectations for patients in routine practice, while also serving as a call for optimizing trial requirements.

The primary finding of this study—the identification of a novel time toxicity EE gap—follows well-established evidence for EE gaps in survival, quality of life, and cost-effectiveness.^12^ The EE gap has largely been attributed to differences in patient factors (trial participants tend to be younger and more fit) and systems factors (trials tend to provide more protocoled, proactive, and equitable care).^11,12^ While these factors synergize to cause and explain the survival EE gap favoring the trial group, they may work in opposite directions when considering time toxicity. Patient-level factors may increase time toxicity in routine practice (patients are less fit and more likely to experience treatment-related toxic effects, resulting in unplanned time toxicity), whereas systems factors (trial requirements) may increase planned time toxicity for trial participants. We observed a clear survival EE gap, which could be explained by differences in patients’ clinical and social backgrounds.^23,24,25,26^

We observed a time toxicity EE gap during systemic treatment. Among patients treated in the first line, trial participants had a longer time on treatment (approximately 4 vs 2 months) and less time toxicity (approximately 19% vs 27%) compared with patients in routine practice. The primary driver of increased contact days in routine practice was inpatient days, with a sharp increase seen in the second half of time on treatment. A systematic review reported higher hospitalization rates for patients with NSCLC in routine practice than in trials (51% vs 16%).^27^ The increase in time toxicity toward the end of treatment may imply that patients in routine practice experience overt clinical progression, whereas patients on trial may discontinue treatment due to earlier radiographic progression. The minisinusoidal on-treatment contact day pattern observed for trial participants may relate to overfitting of the data or that we recorded weekly patterns of contact days (eg, many systemic therapies are administered on an every-3-week schedule), thus creating week-to-week variability.

Previous studies have highlighted the immense time burdens faced by trial participants.^2,3^ Our study did not prospectively compare the time toxicity faced by trial participants vs contemporaneous patients that declined enrollment. We also did not match for patient characteristics that differ between trial patients and routine practice patients. Nonetheless, our results suggest that trial participants did not experience greater time toxicity. We do not propose that trial participation actively decreased time toxicity but are simply comparing experiences for the 2 groups. Reassuringly, trial participants also did not experience delays in initiating treatment. These data serve to allay theoretical concerns about enrolling in trials. Although there was a time toxicity EE gap, it was small overall, and both groups experienced an approximate 20% rate of contact days through the disease course. Recognizing that most patients with advanced NSCLC receiving systemic treatment will spend 1 of every 5 days in contact with the health care system is vital information for patients, regardless of whether they participate in a trial or receive routine treatment. Trial participants experienced mostly outpatient contact days during treatment. Optimizing and minimizing protocol-mandated visits are attractive ways to address trial-related time toxicity. Pragmatic trials are being actively promoted to evaluate the effectiveness of interventions under clinical conditions and supposedly have less stringent investigations and follow-up requirements, which could theoretically reduce time toxicity. Whether pragmatic trials can meaningfully decrease the time toxicities of trials for participants, or whether the expanded eligibility criteria and enrollment of a potentially sicker population reflecting routine practice negates any reductions in time toxicity from changes to trial-mandated procedures, remains to be seen.

### Strengths and Limitations

A key strength of the current study is that both groups received care within the same system (although trial participants had a trial microclimate around them). Most prior work has compared pooled trial data with patient-level data from routine practice.^23,24,25,28^ Trials that lead to drug approvals are often international, with care provided in very different local contexts. The current study overcomes these limitations by comparing experiences within the same health care system. The availability of individual patient data from trials can support more refined EE gap work,^29^ alongside administrative claims data, that can offer longer follow-up.^30^

The study also had some limitations. First, we were unable to investigate drug(s)- or trial-level data due to privacy concerns about reporting on small groups of patients. Second, we lacked detailed contextual clinical information; for example, we did not know whether trial participants additionally received placebo treatments. We did not specifically investigate whether the relevant drug(s) were commonly being used in routine practice. When using recently approved drugs, clinicians may be more cautious and book extra appointments and/or may be less adept at identifying toxic effects early, so patients may present more unwell. Third, by including only decedents and limiting survival and follow-up to 2 years, we might have biased the sample to those with shorter survival. However, given the time taken for adoption in routine practice, a longer survival time would extend care into 2020, with resulting COVID-19–associated changes in care, such as virtual care.

## Conclusions

In this decade-long provincial administrative claims study of patients with advanced-stage NSCLC, we describe a time toxicity EE gap between patients receiving care in routine practice and those in clinical trials. This EE gap was most prominent during the time receiving treatment, when patients in routine practice experienced high rates of hospitalization. These results have ramifications for setting expectations for patients in routine practice. Encouragingly, trial participants did not experience delays in treatment and did not experience greater time toxicity than patients receiving those same treatments in routine practice. The approximate 20% contact day rate for trial participants, mostly composed of outpatient contact days, serves as a call for reevaluating trial requirements.

## Notes

Supplement 1. **eTable 1.** International Classification of Disease O-3 (ICD-O-3) Morphology and Topography Codes for Non–Small Cell Lung Cancer (NSCLC) Diagnoses **eTable 2.** Descriptions of the ICES Health Administrative Databases, Outcomes and Covariates **eFigure 1.** Study Flow Diagram **eFigure 2.** Weekly Contact Days, From Diagnosis to Death

Supplement 2.
Data Sharing Statement
