# Supplementary material for: Health Care Contact Days and Outcomes in Clinical Trials vs Routine Care Among Patients With Non–Small Cell Lung Cancer
Source: JAMA Netw Open. 2025 Apr 15;8(4):e255033. doi: 10.1001/jamanetworkopen.2025.5033 (PMC12000967; doi:10.1001/jamanetworkopen.2025.5033)
Supplement: Supplement 1. — eTable 1. International Classification of Disease O-3 (ICD-O-3) Morphology and Topography Codes for Non–Small Cell Lung Cancer (NSCLC) Diagnoses eTable 2. Descriptions of the ICES Health Administrative Databases, Outcomes and Covariates eFigure 1. Study Flow Diagram eFigure 2. Weekly Contact Days, From Diagnosis to Death [file jamanetwopen-e255033-s001.pdf]

Supplemental Online Content

Gupta A, Nguyen P, Wilson BE, Booth CM, Hanna TP. Health care contact days and outcomes in clinical trials vs routine care among patients with non–small cell lung cancer. *JAMA Netw Open*. Published online April 15, 2025. doi:10.1001/jamanetworkopen.2025.5033

eTable 1. International Classification of Disease O-3 (ICD-O-3) Morphology and Topography Codes for Non–Small Cell Lung Cancer (NSCLC) Diagnoses

eTable 2. Descriptions of the ICES Health Administrative Databases, Outcomes and Covariates

eFigure 1. Study Flow Diagram

eFigure 2. Weekly Contact Days, From Diagnosis to Death

This supplemental material has been provided by the authors to give readers additional information about their work.

© 2025 Gupta A et al. *JAMA Network Open.*

eTable 1: International Classification of Disease O-3 (ICD-O-3) Morphology and Topography Codes for Non–Small Cell Lung Cancer (NSCLC) Diagnoses

ICD-O-3 Code	ICD-O-3 Description
Morphology	
8000/3	Neoplasm, malignant
8001/3	Tumor cells, malignant
8004/3	Malignant tumor, spindle cell type
8010/3	Carcinoma, NOS
8012/3	Large cell carcinoma, NOS
8020/3	Carcinoma, undifferentiated, NOS
8021/3	Carcinoma, anaplastic, NOS
8022/3	Pleomorphic carcinoma
8030/3	Giant cell and spindle cell carcinoma
8031/3	Giant cell carcinoma
8032/3	Spindle cell carcinoma, NOS
8034/3	Polygonal cell carcinoma
8050/3	Papillary carcinoma, NOS
8051/3	Verrucous carcinoma, NOS
8052/3	Papillary squamous cell carcinoma
8070/3	Squamous cell carcinoma, NOS
8070/6	Squamous cell carcinoma, metastatic, NOS
8071/3	Squamous cell carcinoma, keratinizing, NOS
8072/3	Squamous cell carcinoma, large cell, nonkeratinizing, NOS
8073/3	Squamous cell carcinoma, small cell, nonkeratinizing
8074/3	Squamous cell carcinoma, spindle cell
8075/3	Squamous cell carcinoma, adenoid
8076/3	Squamous cell carcinoma, microinvasive
8082/3	Lymphoepithelial carcinoma
8094/3	Basosquamous carcinoma
8120/3	Transitional cell carcinoma, NOS
8130/3	Papillary transitional cell carcinoma
8140/3	Adenocarcinoma, NOS
8140/6	Adenocarcinoma, metastatic, NOS
8141/3	Scirrhous adenocarcinoma
8143/3	Superficial spreading adenocarcinoma
8144/3	Adenocarcinoma, intestinal type
8145/3	Carcinoma, diffuse type
8190/3	Trabecular adenocarcinoma
8200/3	Adenoid cystic carcinoma
8201/3	Cribriform carcinoma, NOS
8210/3	Adenocarcinoma in adenomatous polyp
8211/3	Tubular adenocarcinoma
8230/3	Solid carcinoma, NOS
8231/3	Carcinoma simplex
8250/3	Bronchiolo-alveolar adenocarcinoma, NOS
8251/3	Alveolar adenocarcinoma
8260/3	Papillary adenocarcinoma, NOS
8261/3	Adenocarcinoma in villous adenoma
8263/3	Adenocarcinoma in tubulovillous adenoma
8290/3	Oxyphilic adenocarcinoma
8310/3	Clear cell adenocarcinoma, NOS
8323/3	Mixed cell adenocarcinoma

© 2025 Gupta A et al. *JAMA Network Open.*

8330/3	Follicular adenocarcinoma, NOS
8340/3	Papillary carcinoma, follicular variant
8380/3	Endometrioid carcinoma, NOS
8401/3	Apocrine adenocarcinoma
8410/3	Sebaceous adenocarcinoma
8420/3	Ceruminous adenocarcinoma
8430/3	Mucoepidermoid carcinoma
8440/3	Cystadenocarcinoma, NOS
8441/3	Serous cystadenocarcinoma, NOS
8442/3	Serous cystadenoma, borderline malignancy
8462/3	Papillary serous cystadenoma, borderline malignancy
8470/3	Mucinous cystadenocarcinoma, NOS
8472/3	Mucinous cystadenoma, borderline malignancy
8480/3	Mucinous adenocarcinoma
8481/3	Mucin-producing adenocarcinoma
8490/3	Signet ring cell carcinoma
8490/6	Metastatic signet ring cell carcinoma
8500/3	Infiltrating duct carcinoma
8510/3	Medullary carcinoma, NOS
8550/3	Acinar cell carcinoma
8560/3	Adenosquamous carcinoma
8562/3	Epithelial-myoepithelial carcinoma
8570/3	Adenocarcinoma with squamous metaplasia
8572/3	Adenocarcinoma with spindle cell metaplasia
8802/3	Giant cell sarcoma
8980/3	Carcinosarcoma, NOS
Topography	
C34.0	Main bronchus
C34.1	Upper lobe, lung
C34.2	Middle lobe, lung
C34.3	Lower lobe, lung
C34.8	Overlapping lesion of lung
C34.9	Lung, NOS

Acronyms:
NOS, not otherwise specified

© 2025 Gupta A et al. *JAMA Network Open.*

eTable 2: Descriptions of the ICES Health Administrative Databases, Outcomes and Covariates

Database	Source	Description
Registered Persons Database (RPDB)	MOH	It contains basic demographic information (e.g., age, sex, date of birth, and date of death for deceased individuals) for those issued an Ontario health insurance number.
Ontario Cancer Registry (OCR)	OH	It contains information for all Ontario residents who have been diagnosed with cancer or who have died of cancer.
Ontario Health Insurance Plan (OHIP) Database	MOH	It contains claims and associated diagnoses paid for inpatient and outpatient services (e.g., physicians, and laboratories) provided to Ontario residents eligible for the publicly funded health insurance system.
Discharge Abstract Database (DAD)	CIHI, MOH	It contains administrative (e.g., institution, admission category, length of stay, and disposition), clinical (e.g., diagnoses, and procedures/interventions), and demographic (e.g., age, gender, and location of residence) information for all inpatient admissions to acute care hospitals.
Ontario Mental Health Reporting System (OMHRS)	CIHI, MOH	It contains information on patients in adult designated inpatient mental health beds.
Same Day Surgery (SDS) Database	CIHI, MOH	It contains administrative (e.g., institution, and disposition), clinical (e.g., diagnoses, and procedures/interventions), and demographic (e.g., age, gender, and location of residence) information for all patient visits made to day surgery institutions.
National Ambulatory Care Reporting System (NACRS)	CIHI, MOH	It contains administrative (e.g., institution, and disposition), clinical (e.g., diagnoses, and procedures/interventions), and demographic (e.g., age, gender, and location of residence) information for all patient visits made to hospital- and community-based ambulatory care centers (e.g., emergency departments, hemodialysis units, and cancer care clinics).
Continuing Care Reporting System (CCRS)	CIHI, MOH	It contains clinical (e.g., RAI-MDS 2.0 assessment) and demographic (e.g., primary language spoken, and marital status) information for Ontario residents receiving facility-based continuing care (also known as extended, auxiliary, or complex chronic care) in hospitals or long-term care homes.
National Rehabilitation Reporting System (NRS)	MOH	It contains information on patients in adult designated inpatient rehabilitation facilities and programs.
Ontario Laboratory Information System (OLIS)	MOH	It contains information from an Ontario-wide electronic repository of lab test orders and results from hospitals, community labs and public health labs.
Home Care Database (HCD)	HSSO	It contains information on clients, intake, assessment, admission, diagnostic and surgical procedure, and service delivery who received home care visits.
Cancer Activity Level Reporting (ALR)	OH	It contains information for patient-level activity within the cancer system focused on radiation and systemic therapy services and outpatient oncology clinic visits.
New Drug Funding Program (NDFP)	OH	It contains information for publicly funded high-quality intravenous cancer drugs.
Ontario Drug Benefit (ODB) Program	MOH	It contains prescription medication claims for those covered under the provincial drug program (e.g., residents aged ≥65 years, or receiving social assistance).
Drugs List (DIN)	IQVIA Solutions Canada Inc	It contains drug information on product names, subclass, strength, route of administration, and first and last dispensing dates from the ODB data.

© 2025 Gupta A et al. *JAMA Network Open.*

Symptom Management Database (ESAS)	OH	It contains information from a web-based symptom screening tool for healthcare providers to monitor patient-level symptoms and performance status.
Ontario Asthma (ASTHMA) Database	CIHI, MOH	It identifies Ontario residents with asthma based on an ICES-derived validated algorithm using the OHIP, DAD and SDS data.
Ontario Chronic Obstructive Pulmonary Disease (COPD) Database	CIHI, MOH	It identifies Ontario residents with chronic obstructive pulmonary disease (COPD) based on an ICES-derived validated algorithm using the OHIP, DAD and SDS data.
Ontario Hypertension (HYPER) Database	CIHI, MOH	It identifies Ontario residents with hypertension based on an ICES-derived validated algorithm using the OHIP, DAD and SDS data.
Ontario Congestive Heart Failure (CHF) Database	CIHI, MOH	It identifies Ontario residents with congestive heart failure (CHF) based on an ICES-derived validated algorithm using the OHIP, DAD and SDS data.
Ontario Dementia (DEMENTIA) Database	CIHI, MOH	It identifies Ontario adults aged ≥40 years with dementia based on an ICES-derived validated algorithm using the OHIP, DAD, SDS and ODB data.

Acronyms:
MOH, Ministry of Health; OH, Ontario Health; CIHI, Canadian Institute for Health Information; RAI-MDS, Resident Assessment Instrument Minimum Data Set; HSSO, Health Shared Services Ontario

Descriptions were drawn from the ICES Intranet with adaption from the following references:
1. ICES. ICES data dictionary. https://datadictionary.ices.on.ca. Accessed January 10, 2023. 2023. 2. ICES Privacy & Legal Office. ICES Report to the Information and Privacy Commissioner of Ontario: Three-Year Review as a Prescribed Entity under PHIPA, 2020.

Outcomes

Healthcare contact days were identified with any health administrative records for inpatient acute or rehabilitation hospitalizations (DAD, OMHRS, NRS); emergency department visits (NACRS); outpatient surgeries (SDS); cancer clinic visits (NACRS); dialysis clinic visits (NACRS); long-term or complex continuing care (CCRS); outpatient contact (e.g., family physician, imaging, injections/infusions, and radiotherapy assessment and treatments) (OHIP); and blood lab test visits (OLIS) from the selected ICES databases (in bold). Healthcare contact was counted once per day regardless of the length, reason or cause of contact.

Covariates

Socioeconomic status was based on community-specific or neighborhood household income quintiles. Rurality of residence was classified using the 2008 Rurality Index for Ontario (RIO), with higher scores representing a greater degree of rurality: Rural area (RIO score ≥40), suburban area (10≤ RIO score <40), and urban centers (RIO score <10).

Comorbidities were measured using the Elixhauser comorbidity index derived from hospital records from the DAD and SDS databases with a 5-year lookback from their NSCLC diagnosis. Chronic conditions (e.g., asthma, hypertension, and dementia) were based on the ICES-derived databases.

Systemic therapy was described with specific anticancer medications from the ALR, NDFP and ODB databases, while radiotherapy was defined with specific treatment activities from the ALR database. Metastasis surgeries were identified from cancer-related surgical interventions from the DAD database related to brain resection or spinal cord compression. Palliative systemic therapy, radiotherapy and metastasis surgery treatment were measured from NSCLC diagnosis to death.

© 2025 Gupta A et al. *JAMA Network Open.*

eFigure 1: Study flow diagram

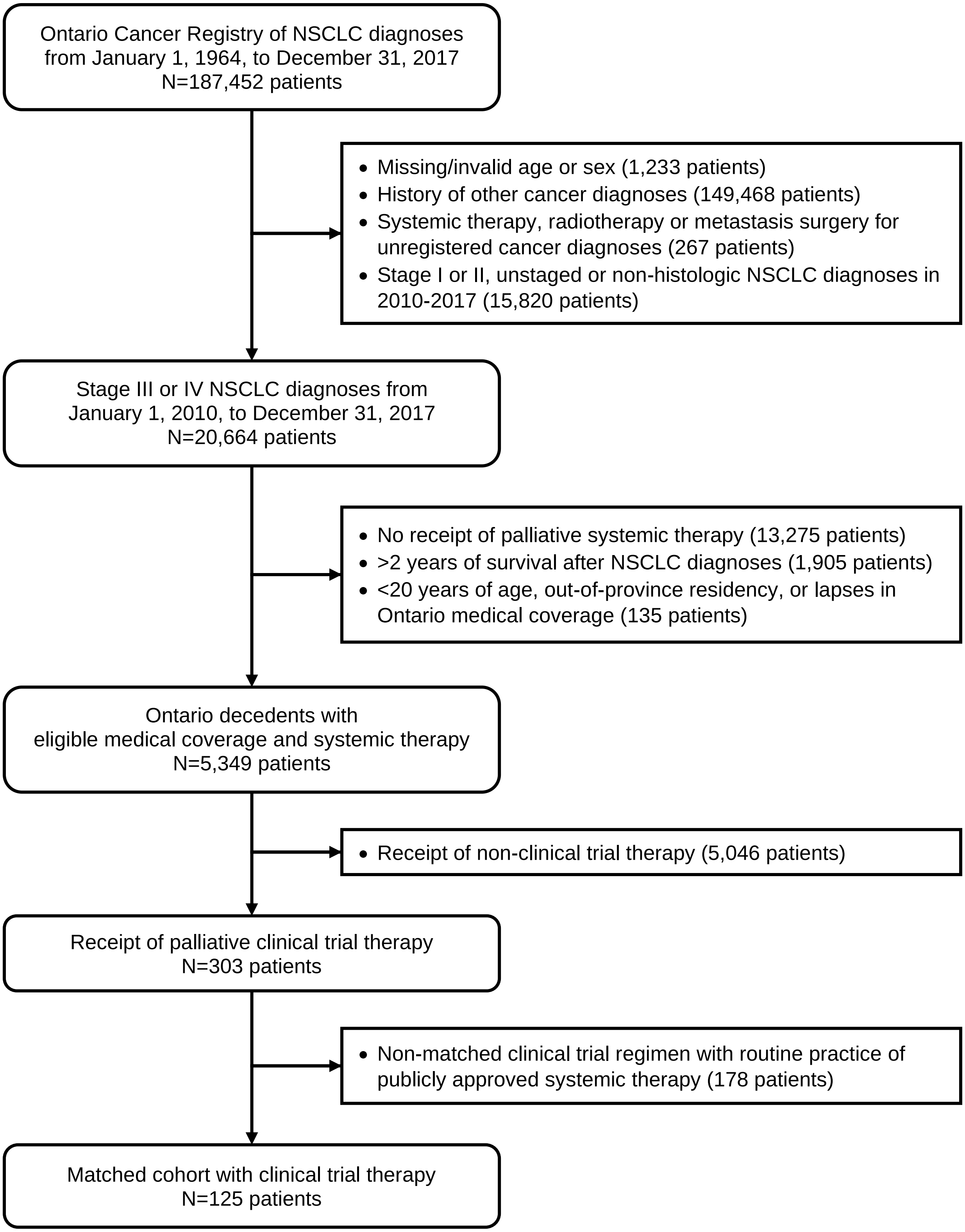

© 2025 Gupta A et al. *JAMA Network Open.*

eFigure 2: Weekly contact days, from diagnosis to death:

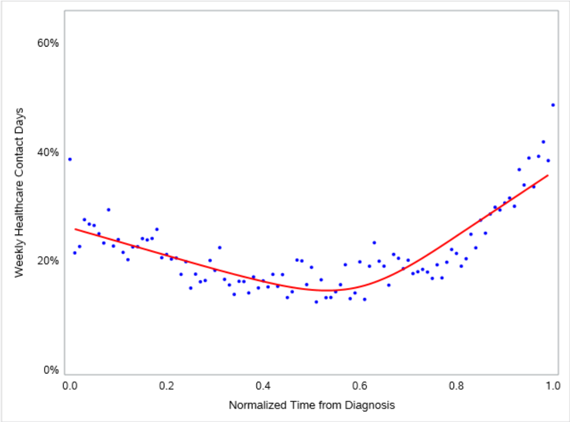

a) Trial patients, total contact days

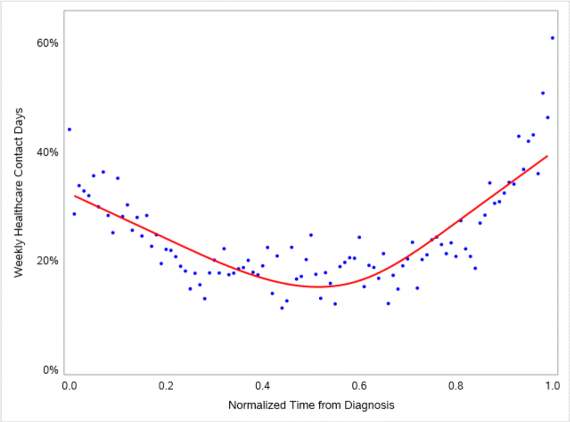

b) Patients in routine practice, total contact days

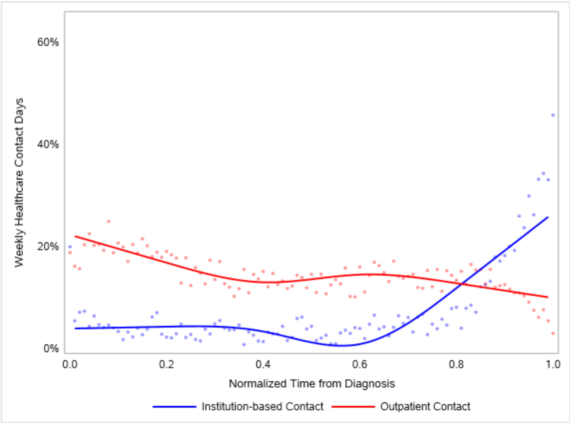

c) Trial patients, inpatient and outpatient contact days

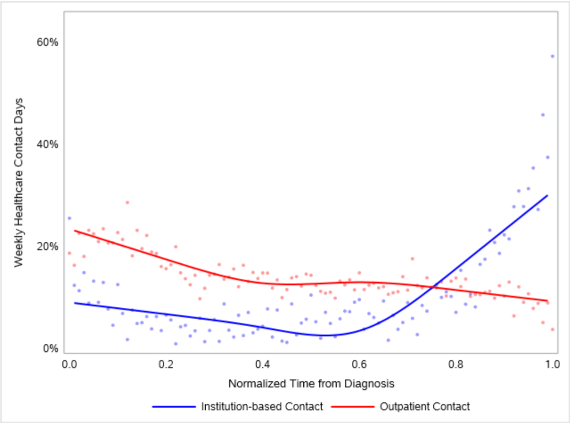

d) Patients in routine practice, inpatient and outpatient contact days

© 2025 Gupta A et al. *JAMA Network Open.*
